# Development and Implementation of a Pilot Registry for Monitoring the Efficacy and Safety of Novel Therapies in Patients with Systemic Lupus Erythematosus

**DOI:** 10.31138/mjr.31.1.87

**Published:** 2020-03-31

**Authors:** Christina Adamichou, Irini Flouri, Antonios Fanouriakis, Myrto Nikoloudaki, Dionysios Nikolopoulos, Argyro Repa, Kyriaki Boki, Katerina Chatzidionysiou, Alexandros Garyfallos, Dimitrios Boumpas, Prodromos Sidiropoulos, George Bertsias

**Affiliations:** 1 Rheumatology and Clinical Immunology, University of Crete Medical School and University Hospital of Heraklion, Heraklion, Greece,; 2 Rheumatology Clinic, General Hospital of Athens “Asklepeion Voula”, Athens, Greece,; 3 Rheumatology Clinic, 4^th^ Department of Internal Medicine, National and Kapodistrian University of Athens, “Attikon” University Hospital, Athens, Greece,; 4 Rheumatology Clinic, “Sismanogleio” General Hospital, Athens, Greece,; 5 First Department of Propaedeutic Internal Medicine, National and Kapodistrian University of Athens, “Laiko” General Hospital, Athens, Greece,; 6 Department of Medicine, Solna, Rheumatology Unit, Karolinska University Hospital and Institutet, Stockholm, Sweden,; 7 4^th^ Department of Internal Medicine, Hippokration General Hospital, School of Medicine, Aristotle University of Thessaloniki, Thessaloniki, Greece

**Keywords:** registry, biological agents, novel therapies, belimumab, rituximab, systemic lupus erythematosus

## Abstract

The therapeutic armamentarium in Systemic Lupus Erythematosus (SLE) is expanding with the introduction of novel biologic and small-molecule agents. Complementary to randomized controlled trials, registry-based studies are advantageous due to the inclusion of a wider range of patients from daily practice and the potential for long-term monitoring of the efficacy and safety of therapies. Moreover, data from registries can be used to identify disease phenotypes that best respond to biologic agents, and to correlate clinical response with parameters such as co-administered therapies and comorbidities. In this project, we will use the configuration of the Hellenic Registry of Biologic Therapies for inflammatory arthritides in order to design a dedicated SLE module with variables pertaining to global and organ-specific disease activity, severity, flares, organ damage/outcome, comorbidities and adverse events. The second stage will involve the pilot implementation of this platform for the multicentric registration of SLE patients who are treated with belimumab. The significance lies in the development of a structured registry that enables the assessment of the disease burden and the long-term efficacy and safety of existing and future biological agents in SLE. Piloting the registry can serve as a basis for establishing nationwide collaborative efforts.

## BACKGROUND AND STUDY RATIONALE

Systemic lupus erythematosus (SLE) encompasses a wide range of clinical and immunological manifestations, which makes monitoring of patients and assessment of their response to therapy a challenging task.^1^ For many years, treatment of SLE was based primarily on the administration of corticosteroids and non-specific immunomodulators/suppressors or cytotoxic agents. Although these agents are generally efficacious in controlling the disease, still, a considerable proportion of patients fails to achieve long-standing remission.^2^ Importantly, conventional drugs, particularly corticosteroids, are associated with excessive toxicity risks, accrual of comorbidities and irreversible end-organ damage.^3^

Scientific advancements in our understanding of the immunopathogenesis of SLE, coupled with better-designed clinical studies,^4^ have led to the expansion of the therapeutic armamentarium due to repositioning of drugs administered in other medical conditions (eg, mycopheno-late^5^) and the increasing use of approved (e.g. belimumab^6^) and non-approved (eg, rituximab^7^) biologic agents. Based on the findings of recently performed controlled trials,^8– 14^ a number of innovative therapies in SLE, including monoclonal antibodies (eg, anifrolumb, ustekinumab, obinutuzumab) and small molecules (eg, Janus kinase inhibitors), are expected to be introduced in the near future.

Post-marketing analysis of the efficacy and safety of new drugs is important in defining their application in routine clinical practice. In addition to the results from randomized clinical trials (RCTs), a significant amount of information can be derived from patient registries that systematically assess the effects of treatments under “real-life” conditions. Registries are also advantageous because they allow the inclusion of a wide spectrum of patients without the stringent exclusion criteria of RCTs (eg, patients with co-morbidities or less frequent disease manifestations), and monitoring for long-term drug efficacy and safety (including rare adverse events).^15,16^

In the case of SLE, which is a multifaceted, systemic auto-immune disease, data from organized patient registries may be particularly useful to approach clinically-relevant issues that cannot be easily addressed through clinical trials. These may include, for instance, the definition of disease endo-phenotypes that respond better to individual treatments, and the association between drug efficacy/safety with various disease parameters (co-administered treatments, comorbidities, etc.). Furthermore, collected data may help to obtain unique insights into the mechanisms of action of novel therapies.

The value of establishing registries of patients receiving biological agents has been illustrated in inflammatory arthritides (rheumatoid arthritis, spondyloarthritis). Thus, analysis of registry-derived data has shed light on topics such as the identification of clinical factors that are predictive of treatment response, the safety of biologics in terms of the risk for latent infections and malignancies, the use of sequential therapies (‘switches’), the main causes of drug discontinuations and other.^17–20^ Likewise, large SLE registries, such as the Johns Hopkins Lupus Cohort, have provided significant knowledge with regards to the dose-dependent corticosteroid toxicity and the role of hydroxychloroquine in prevention of disease flare-ups.^1,3^ Another example is the British Isles Lupus Assessment Group (BILAG) Biologics Register of SLE patients, which has described the main features of patients who are candidates for biological treatment and has investigated the short-term efficacy and safety associated with the use of rituximab.^7^

To date, there is no structured platform for registering SLE patients in Greece under biologic therapies, and, accordingly, there is paucity of data on the indications, long-term efficacy and safety of these agents in real-life clinical settings. In this study, we seek to establish and implement a pilot system for the electronic registration and monitoring of patients with SLE who are treated with existing biologic but also, future novel therapeutics agents.

## AIMS OF THE STUDY

The aim of the present study is to establish and run a pilot study of an electronic registry for monitoring SLE patients who are treated with novel/biologic therapies. The study has multicentric, prospective design with two implementation stages.

## METHODS

### Study design

This is a prospective study that will be performed at the Rheumatology Clinic, University of Crete Medical School and University Hospital of Heraklion (involved at stages I and II of the protocol) in collaboration with the Rheumatology Units/Clinics of the “Attikon” University Hospital, “Laiko” General Hospital, General Hospital of Asklepieion Voula, Hippokration University Hospital of Thessaloniki, and “Sismanogleio” General Hospital (involved at stage II of the protocol). The study has been approved by the Ethics Committees of the participating centers.

The first stage (0–6 months) of the protocol includes the design of a specialized electronic platform (registry) for patients with SLE. The development of the registry will be based on the configuration and software of the existing registry of patients with chronic inflammatory arthritides who are treated with biologic agents (University of Crete, Medical School),^17^ following amendments to capture at each visit: the dosage of main and secondary treatments, general and organ-specific indices of SLE disease activity, flares, organ damage, health-related quality of life and functional status, comorbidities, adverse events and causes of drug discontinuation (outlined below).

The second stage (7–30 months) of the protocol will include the pilot use of the electronic registry for the multicentric enrolment and longitudinal monitoring of patients who meet the following inclusion criteria: a) fulfil at least one of the three classification criteria (ACR 1997,^21^ SLICC 2012,22 EULAR/ACR 201923) for SLE, and b) already receive or are started on belimumab, which represents the single approved biologic agent in SLE.^24^

We also plan to update the long-term efficacy and safety data of a previously published cohort of patients.^6^ We will assess the effectiveness (both globally and across individual organs/domains), attainment of low disease activity and remission states, the co-administration of glucocorticoids and other treatments, major events (comorbidities) and adverse events (outlined below).

### Design of the electronic registry

An online software is under development at the Rheumatology Clinic, University of Crete Medical School, in collaboration with the Centre for eHealth Applications and Services (CeHA) at the Institute of Computer Science, Foundation for Research and Technology – Hellas (ICS-FORTH) (http://web-new.ics.forth.gr/ceha/). This is a web-based platform with secure connection, anonymised entry of patient data, encrypted data storage in line with the General Data Protection Regulation (GDPR) and ability to export selected data for further analysis. The SLE registry module will be based on the structure and configuration of the Hellenic Registry of Biologic Therapies for patients with inflammatory arthritides.^17^

### Registry variables

From each patient, the following variables will be collected at inclusion visit (initiation of treatment) and/or at regular (6-month) follow-up intervals:

- Demographics (gender, nationality, date of birth)
- Date of diagnosis
- SLE classification criteria (ACR 1997,^21^ SLICC 2012,22 EULAR/ACR 2019^23^)
- Disease stratification (mild, moderate, severe) based on BILAG-defined organ activity,^25,26^ administration of corticosteroids and use of potent immunosuppressive/cytotoxic or biologic treatments
- Disease activity pattern (relapsing-remitting, chronic active)
- Global activity indices (SLEDAI-2000,^27^ Physician Global Assessment [PhGA]^28^)
- Organ-specific activity indices (CLASI index for cutaneous lupus,^29^ tender and swollen joint counts, proteinuria, Likert scale for neurological deficits)
- Disease flares (SELENA-SLEDAI Index^28^)
- Definitions of Lupus Low Disease Activity State (LLDAS)^30^ and remission^31^
- Irreversible organ damage (SLICC/ACR Damage Index^32^)
- Functional status (Health assessment questionnaire disability index [HAQ-DI]^33^)
- Comorbidities (Rheumatic Disease Comorbidity Index [RDCI]^34^)
- Treatments (previous, ongoing): detailed record of medications (main treatment, concomitant disease treatments) and administered forms/dosage
- Adverse reactions and events (MedDRA recording system; https://www.meddra.org/faq/meddra-general), treatment discontinuations

### Data entry and collection

Each participating centre will be granted access to the electronic platform (registry) with unique credentials in order to enter their own patient data. As this is a pilot implementation of the registry, we aim to enrol a total of 60 SLE patients under treatment with belimumab, which will allow to obtain statistically robust results.

### Statistical analysis

A single export of merged patient data from all centres will be obtained for statistical analysis (months 31–36). Descriptive results on demographics, proportion of patients with partial or complete clinical response, attainment of LLDAS and remission/flares will be calculated. Efficacy will be correlated with baseline clinical and demographic characteristics. Safety will be assessed according to occurrence of major events. Treatment survival and reasons for discontinuation will also be evaluated.

## ANTICIPATED RESULTS AND PROJECT SIGNIFICANCE

Registries of patients with chronic rheumatic diseases receiving biological therapies have provided significant insights regarding their long-term efficacy and safety in “real-life” clinical practice. In recent years, biological agents such as belimumab and rituximab have been introduced in the treatment of SLE, and based on the results from ongoing clinical trials, it is likely that additional innovative therapies may be added in the near future. The importance of this project lies in the development and pilot use of a dedicated electronic registry of patients with SLE who are treated with novel/biologic therapies, which will enable the detailed monitoring of drug efficacy and safety by the use of validated clinical instruments (eg, SLEDAI-2000, CLASI, RDCI, MedDRA). Moreover, the registry will help to assess the burden of the disease (severe lupus, flares, organ damage, comorbidities) among contemporary SLE patients who are seen at large Rheumatology Centres in Greece, as well as the long-term retention rates of the biological agents used in SLE such as belimumab. Importantly, the successful pilot implementation of the registry could pave the way for establishing broader collaborative projects at a national level.
